# The Effect of Zeolite Composition and Grain Size on Gas Sensing Properties of SnO_2_/Zeolite Sensor

**DOI:** 10.3390/s18020390

**Published:** 2018-01-29

**Authors:** Yanhui Sun, Jing Wang, Xiaogan Li, Haiying Du, Qingpan Huang, Xiaofeng Wang

**Affiliations:** 1 Faculty of Electronic Information and Electrical Engineering, Dalian University of Technology, Dalian 116024, China; syh@dlnu.edu.cn (Y.S.); duhaiying@dlnu.edu.cn (H.D.); huangqingpan@mail.dlut.edu.cn (Q.H.); 2 College of Information & Communication Engineering, Dalian Minzu University, Dalian 116600, China; 3 College of Mechanical and Electronic Engineering, Dalian Minzu University, Dalian 116600, China; 4 School of Mathematical and Physical Sciences, Dalian University of Technology, Panjin Campus, Panjin 124000, China; wangxf@dlut.edu.cn

**Keywords:** gas sensor, zeolite, tin dioxide, selectivity, catalytic property, coating

## Abstract

In order to improve the sensing properties of tin dioxide gas sensor, four kinds of different SiO_2_/Al_2_O_3_ ratio, different particle size of MFI type zeolites (ZSM-5) were coated on the SnO_2_ to prepared zeolite modified gas sensors, and the gas sensing properties were tested. The measurement results showed that the response values of ZSM-5 zeolite (SiO_2_/Al_2_O_3_ = 70, grain size 300 nm) coated SnO_2_ gas sensors to formaldehyde vapor were increased, and the response to acetone decreased compared with that of SnO_2_ gas sensor, indicating an improved selectivity property. The other three ZSM-5 zeolites with SiO_2_/Al_2_O_3_ 70, 150 and 470, respectively, and grain sizes all around 1 μm coated SnO_2_ sensors did not show much difference with SnO_2_ sensor for the response properties to both formaldehyde and acetone. The sensing mechanism of ZSM-5 modified sensors was briefly analyzed.

## 1. Introduction

Formaldehyde is an important chemical industrial material and organic solvent and it is widely used in wood processing, the textile industry and in other fields. Formaldehyde is also a kind of harmful gas. Excessive inhalation of formaldehyde in the body can cause discomfort in the eyes and nose. Prolonged time spent in a formaldehyde environment will lead to the occurrence of cancer. A standard of 0.08 ppm averaged over 30 min for long-term exposure in formaldehyde vapor has been established by the World Health Organization (WHO) [1]. In recent years, more and more bodily harm occurs because of the excessive levels of formaldehyde which exist in the renovation materials and furniture. The monitor and control of indoor air pollution are attracting much attention recently [2,3,4,5]. Conductivity measurement using the semiconductor gas component is a common method of gas concentration detection. Gas sensors made of metal oxides possess good sensing properties such as high sensitivity, short response and recovery times and low cost. Tin dioxide is a kind of common metal oxide gas sensing material. SnO_2_ gas sensor can react with the electron of the gas, then the electrons are bound by the adsorbed oxygen. This decreases the conduction electrons in surface space-charge region of N-type material, so the resistance of the material is high. When tin dioxide meets reducing gas, the gas will react with adsorbed oxygen and release the oxygen-binding electronic. As a result, the surface electrical conductivity increases and the resistance of the material decreases [6]. Because the SnO_2_ has an adsorption capacity to many organic gases, the poor selectivity is an inherent problem. The SnO_2_ sensor has good response for many VOCs gases, such as methanol [7], ethanol [8], formaldehyde [9], acetone [10], etc. A lot of research has focused on how to improve the selectivity of gas sensors. Various methods including temperature control, doping noble metal and addition of filter layer may be used to improve sensor selectivity [11,12,13,14,15,16]. Among them, the addition of filter layer is the lowest cost and most convenient method.

Zeolites are kinds of aluminosilicates crystalline materials. They are built up with SiO_4_ and AlO_4_ tetrahedra, which form three-dimensional (3D) frameworks with linked channel systems and well-defined micropores and mesopores [17,18]. Zeolites possess molecular sieve property; they can make the gas molecules with smaller size pass through the zeolites channels and block the bigger molecules [19]. Utilizing the zeolites as a physical filter layer, zeolites can effectively improve selectivity of some semiconductor gas sensors. Many researches have focused on the application of zeolite as a physical filter layer in the sensitive material. For example, thick film sensors were prepared by screen printing layers of chromium titanium oxide (CTO) and tungsten trioxide with A-type zeolite, Y-type zeolite and ZSM-5 zeolite as over-layers, respectively, and the target gases were two similar gases: ethanol and isopropyl alcohol. The response of the CTO/H-A sensor to isopropyl alcohol (IPA) was suppressed by the A-type zeolite layer because of the molecular sieving effect [20]. Similarly, the WO_3_ and H-ZSM-5 composites can be printed onto an aluminum substrate to enhance the selectivity of NO_2_ [21]. Micromachined sensors was prepared by A-type zeolite microdropping on the Pd doped SnO_2_ sensitive material. The measurement results showed that the responses for some gases were suppressed, such as CO, H_2_, CH_4_, C_3_H_8_, but the response for ethanol was not changed [22]. The selectivity of SrTi_1−*x*_Fe*_x_*O_3−*δ*_ sensor to propane was improved by zeolite Pt-MFI modified [23]. The selectivity of La_2_O_3_–Au/SnO_2_ sensor to CO was improved after it was modified using zeolite FER [24].

Zeolites possess both molecular sieve and catalytic properties. Normally, the molecular sieve property of zeolite was used to improve the selectivity of composite materials [25,26], and the catalytic property was often used to increase the response to target gas [15,27]. It is generally believed that the process of target gas molecules passing through the zeolite layer to the sensitive material is as follows [15,28]: (1) The target gas diffused into the pore channel of the zeolite and reached the catalytic active sites; (2) The gas molecules were adsorbed on the catalytic active sites (acidic sites); (3) A series of catalytic reactions occurred between the gas molecules and the acid sites of the zeolite and produced the catalytic by-products; (4) The reaction products deactivated from the active sites and spread out from the zeolite channel, and then reached to the surface of the sensitive material. In this process, the number of catalytic active sites (acidic sites) in zeolite plays an important role. In general, the number of zeolitic acidic sites decreases with the increase of SiO_2_/Al_2_O_3_ ratio. The reduction of zeolite grain size also leads to an increase in the zeolite specific surface area and pore volume. However, the increase of pore volume will further increase its acidic sites and thus increase its catalytic activity [29,30].

Based on the above considerations, this report focuses on the effect of the gas sensing characteristics about different SiO_2_/Al_2_O_3_ ratio and different grain size of ZSM-5 as the adding layer on SnO_2_ sensors to improve the sensor’s sensing properties. The research about the effect of the grain size of the zeolite on the gas sensing properties were rarely reported in previous literature. In addition, numerous research has focused on the detection of CO, H_2_, NO_2_ and other target gases [21,24,31,32], and there were few reports about the research of detecting formaldehyde using this type of gas sensors.

## 2. Experimental

### 2.1. ZSM-5 Zeolite Preparation

Four kinds of ZSM-5 zeolites have been used in this study, small particle size, low SiO_2_/Al_2_O_3_ ratio of ZSM-5 zeolite (named MFI-S); SiO_2_/Al_2_O_3_ ratio of ZSM-5 zeolites were 70, 150 and 470 (named MFI-70, MFI-150 and MFI-470), respectively. The latter three kinds of zeolites were purchased from the Catalyst Plant of Nankai University, Tianjin, China. The MFI-S zeolite was prepared by hydrothermal synthesis following template method in our laboratory [33]. Silica source (TEOS) and NaOH purchased from Tianjin Kemiou Chemical Reagent Co., Ltd. (Tianjin, China), aluminum source (aluminum isopropoxide) purchased from Tianjin Guangfu Fine Chemical Research Institute (Tianjin, China), template agent (TPAOH) purchased from Sinopharm Chemical Reagent Co., Ltd. (Shanghai, China), respectively. The typical synthesis procedure was as follows: The predetermined amount of deionized water was added into 25% TPAOH aqueous solution to make a 15.7% TPAOH aqueous solution (50 g), which was stirred with deionized water (16 g) and 1.12 g NaOH for 10 min till the solution became clear. Then, 0.27 g aluminum isopropoxide was poured into the clear solution and kept stirring for 5 h to get transparent solution. After that, 32 mL TEOS was mixed with the transparent solution and kept stirring 12 h and the resulting mixture was transferred into a Teflon-lined stainless-steel autoclave and heated at 100 °C for 12 h. Then the temperature increased to 180 °C and crystallization continued for 12 h. The resultant solid product was separated from the mother liquor by centrifugation. After cooling, the mixture was centrifuged at 4000 rpm for 30 min to discard the supernatant. The predetermined amount of deionized water was added into the centrifuge tube and made the solid disperse in the deionized water by ultrasonic vibration for 10 min. The above procedure was repeated twice. Then, the sample was washed again with ethanol. Afterwards, the samples were dried at 100 °C for 2 h followed by calcination at 550 °C for 6 h. The temperature rising rate was 2 °C/min.

### 2.2. Characterization

The structures of the ZSM-5 zeolites were characterized by an X-ray diffraction instrument (XRD: D/Max 2400, Rigaku, Japan) in 2θ region of 3–60° at a rate of 6°/min with Cu Kα radiation. The morphology images of ZSM-5 zeolites and cross-sectional SEM image of ZSM-5 coated SnO_2_ sensor were obtained by using field emission scanning electron microscope (FE-SEM: Hitachi S-4800, Tokyo, Japan). The ratio of SiO_2_/Al_2_O_3_ for the four kinds of ZSM-5 zeolites were measured by Coupled plasma atomic emission spectrometer (ICP-OES: Optima 2000 DV, PerkinElmer, Waltham, MA, USA).

### 2.3. Sensors Fabrication and Measurements

The SnO_2_ were mixed with deionized water to form a paste. The paste was coated onto a clean ceramic tube with electrodes and wires to form a sensing film, and then annealed at 400 °C for 2 h in air. Four kinds of ZSM-5 zeolite were also mixed with deionized water to form pastes, respectively, and coated outside of the SnO_2_ sensors to form four kinds of ZSM-5 coated SnO_2_ gas sensors (MFI-S, MFI-70, MFI-150 and MFI-470). The final ceramic tube was annealed at 400 °C for 2 h in air, and then a Ni-Cr resistor wire was threaded through the tube as a heater. Finally, the electrode and heater wires were welded on a base element to form inside-heated gas sensors. The SnO_2_ sensor used for comparison with ZSM-5 coated SnO_2_ sensors in this work was fabricated by the above method. The SnO_2_ powder was purchased from Sinopharm Chemical Reagent Co., Ltd. The SnO_2_ nanoparticles were relatively uniform with average grain size of ~10 nm.

**Figure 1:**
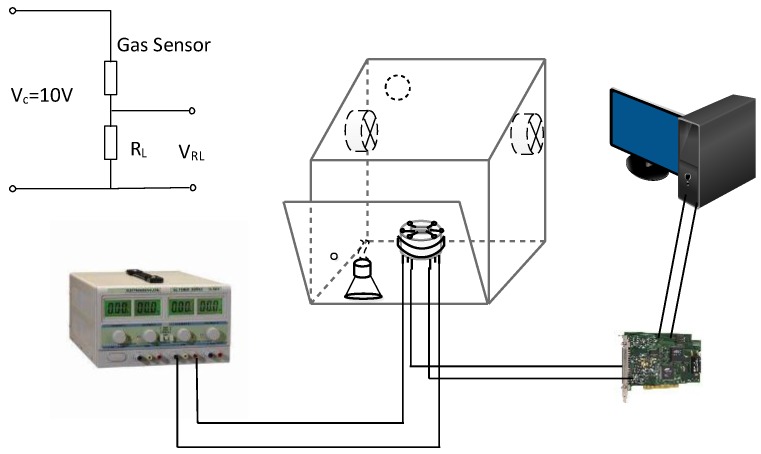
Structure diagram of gas sensing characterization system.

The static state gas sensing characterization system was used to measure the gas sensing properties of the above sensors. The structure diagram of the static state gas sensing characterization is shown in Figure 1. The gas sensors were placed in a test chamber with a volume of 50 L. A given amount of target gas solution was injected into a heated crucible in the test chamber by micro injector, then, it was uniformly distributed throughout the test chamber driven by two fans. In a gas desorption process, the test chamber was opened and the element was exposed to the air. The export voltage of the gas sensor is measured by a voltage dividing circuit that realized by series of gas sensor and divider resistance *R*_L_. The test voltage loaded at the voltage dividing circuit is 10 V. By replacing the divider resistance *R*_L_, the beginning voltage (*V*_air_) of the divider resistor *R*_L_ is controlled at 8–9 V, and the voltage of the gas sensor is 1–2 V. According the resistance value of divider resistance *R*_L_ and the voltage value of the divider resistance (*V*_gas_), the sensor response (*S*) to the test gas is calculated as *S* = *R*_a_/*R*_g_, and *R*_a_ = *R*_L_ (10 − *V*_air_)/*V*_air_, *R*_g_ = *R*_L_ (10 − *V*_gas_)/*V*_gas_. Where *R*_a_ and *R*_g_ are resistance of the sensor in air and in target gas, respectively [34].

## 3. Results and Discussion

### 3.1. Materials Characterization

**Figure 2:**
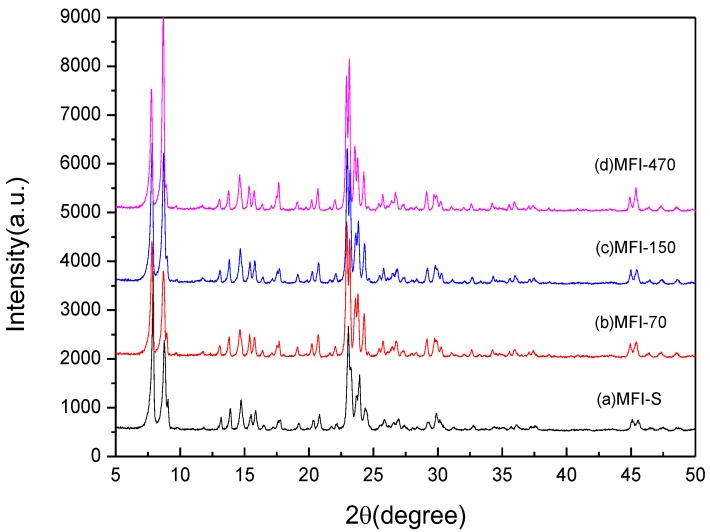
XRD patterns of (**a**) MFI-S (**b**) MFI-70 (**c**) MFI-150 and (**d**) MFI-470.

The X-ray diffraction patterns (XRD) of the zeolites that used in the study are shown in Figure 2. The XRD of the samples showed the peaks corresponding to the ZSM-5 type zeolite. These four kinds of materials have the same characteristic peaks, indicating that the four materials have the same structure.

**Figure 3:**
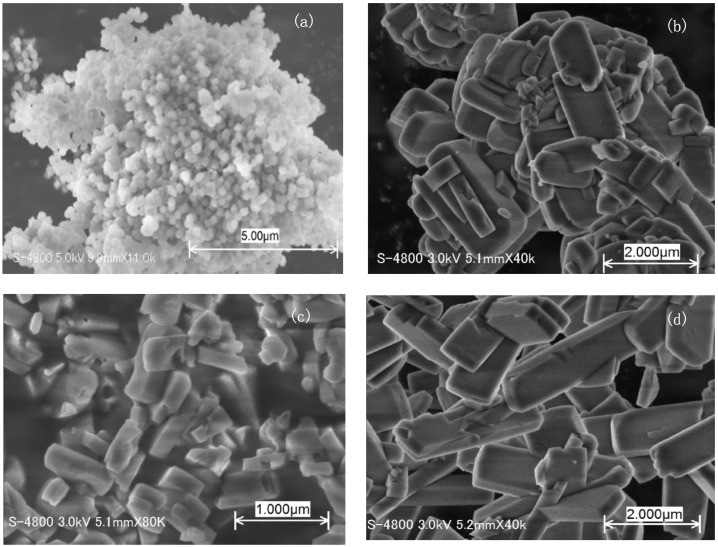
SEM images of (**a**) MFI-S; (**b**) MFI-70; (**c**) MFI-150 and (**d**) MFI-470.

**Figure 4:**
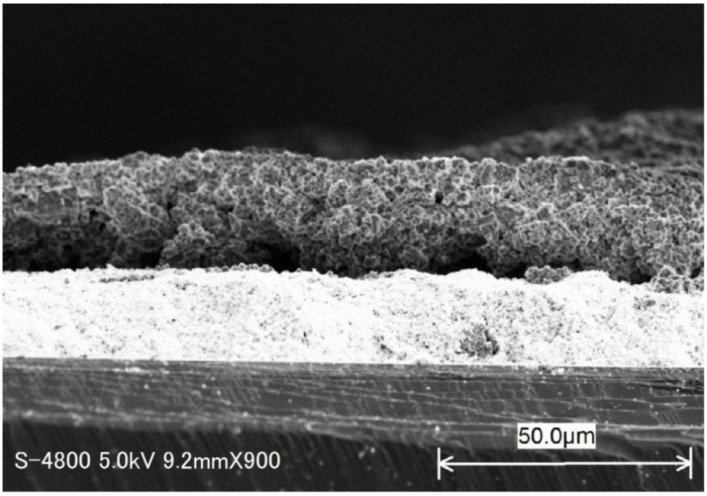
Cross-sectional SEM image for ZSM-5 coated SnO_2_ sensor.

Figure 3a–d gives the SEM images of the four kinds of ZSM-5 zeolite samples. We can see from Figure 3 that the zeolites crystal lines are relatively uniform and the surfaces of the particles are relatively smooth. The average grain size of MFI-S (300 nm) is much smaller than these of other three samples (~1 μm). The average grain size of the latter three samples are nearly the same. Figure 4 shows the cross-sectional SEM image for ZSM-5 coated SnO_2_ sensor. The thicknesses of SnO_2_ sensitive and ZSM-5 coated layers are around 15 μm and 20 μm, respectively.

The ratio of SiO_2_/Al_2_O_3_ for the four kinds of ZSM-5 zeolites were measured by Coupled plasma atomic emission spectrometer. Table 1 lists the SiO_2_/Al_2_O_3_ ratio and the grain size of four kinds of zeolite. It can be seen from Table 1 that the SiO_2_/Al_2_O_3_ ratio of MFI-S is the same as the MFI-70, but the grain size is quite different. The grain size of MFI-70, MFI-150 and MFI-470 are similar, but the ratio of SiO_2_/Al_2_O_3_ increases gradually.

### 3.2. Gas Sensing Properties

**Figure 5:**
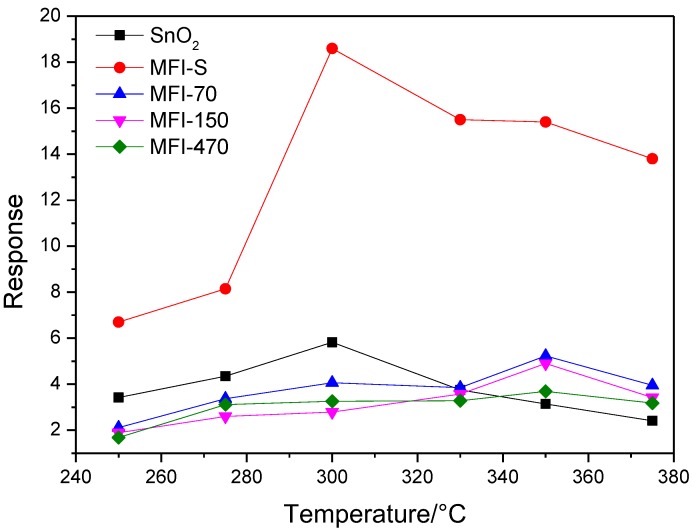
Relationships between sensors response and operating temperature.

Operating temperature is an important parameter for semiconductor gas sensor. Figure 5 illustrates the responses of the SnO_2_ gas sensor and four kinds of ZSM-5 zeolites coated SnO_2_ gas sensors to 10 ppm formaldehyde vs. operating temperature ranged from 250 to 380 °C. The relative humidity (RH) is about 40% RH. We can see from Figure 5 that the maximum response value to 10 ppm formaldehyde for different sensors appeared at different temperatures. The optimum operating temperature are 350 °C for MFI-70, MFI-150 and MFI-470 coated SnO_2_ sensors, and the temperature is slightly higher than that of MFI-S coated SnO_2_ sensor and SnO_2_ sensor (300 °C). The reasons for these two different optimum operating temperatures may be related to the grain size of zeolites ZSM-5. The heterogeneous catalytic temperature of zeolite has a certain relation with grain size [35]. The smaller the grain size, the lower the catalytic temperature and vice versa. As the grain size of the MFI-S (~300 nm) is much smaller than that of the other three zeolites (~1 μm), so that the optimal operating temperature of the MFI-S coated SnO_2_ sensor is slightly lower than the latter three.

Figure 5 shows that the maximum response value to 10 ppm formaldehyde of MFI-S coated SnO_2_ gas sensor (18.6) is higher than that of SnO_2_ gas sensor (5.8). The optimum operating temperature is 350 °C for MFI-70, MFI-150 and MFI-470 coated SnO_2_ sensors, and the temperature is slightly higher than that of MFI-S coated SnO_2_ sensor and SnO_2_ sensor.

**Figure 6:**
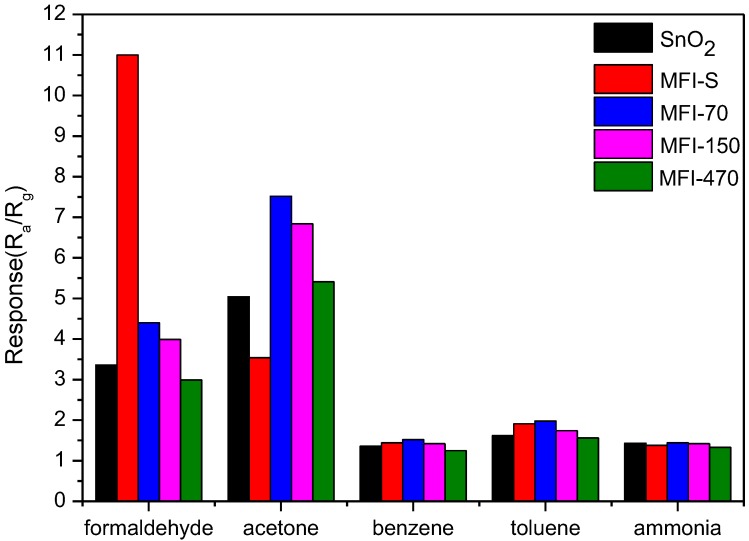
Responses of ZSM-5 coated SnO_2_ sensors and SnO_2_ sensors to different gases in 45% RH.

The responses of ZSM-5 coated SnO_2_ sensors to 10 ppm different VOCs gases are plotted together and compared to the SnO_2_ sensors’ in the bar diagram presented in Figure 6. The VOCs gases include formaldehyde, acetone, toluene, benzene and ammonia. The measurement results show that the ZSM-5 coated SnO_2_ sensors have great influence on the responses of formaldehyde and acetone, but have little effect on the responses of toluene, benzene and ammonia. The response values to formaldehyde and acetone are significantly higher than that of toluene, benzene and ammonia, respectively. Table 2 gives the response values of four kinds of ZSM-5 coated SnO_2_ sensors and SnO_2_ sensor to 10 ppm formaldehyde, acetone, toluene, benzene and ammonia with a relative humidity 45% RH, respectively. We find that compared with the SnO_2_ sensor, the response of MFI-S coated sensor to formaldehyde increased from 3.5 to 11.0, but acetone decreased from 5.0 to 3.5. However, for ammonia, toluene and benzene vapors, the “coating effect” is not obvious and remains at a relatively low response value (less than 2). This indicates that MFI-S coating indeed improves the selectivity of SnO_2_ gas sensor to formaldehyde against acetone, toluene, benzene and ammonia vapors. Further analysis of the data in Figure 6 reveals that the responses of the other three ZSM-5 zeolites (MFI-70, MFI-150, and MFI-470) coated SnO_2_ sensors to formaldehyde change a little. Although SiO_2_/Al_2_O_3_ ratio of the three samples are different, the grain sizes are nearly the same (~1 μm). This indicates that the grain size plays more important role than SiO_2_/Al_2_O_3_ ratio here. We can also see from Figure 6 that the response of the MFI-S coated sensor to acetone decreases and the other three ZSM-5 zeolite coated sensors increase in a small scale compared with the SnO_2_ sensor. Comparing the responses of SnO_2_ and MFI-S coated SnO_2_ gas sensors to formaldehyde and acetone, we can conclude that the interference from acetone was very little when formaldehyde was detected by using the MFI-S coated SnO_2_ sensor.

**Figure 7:**
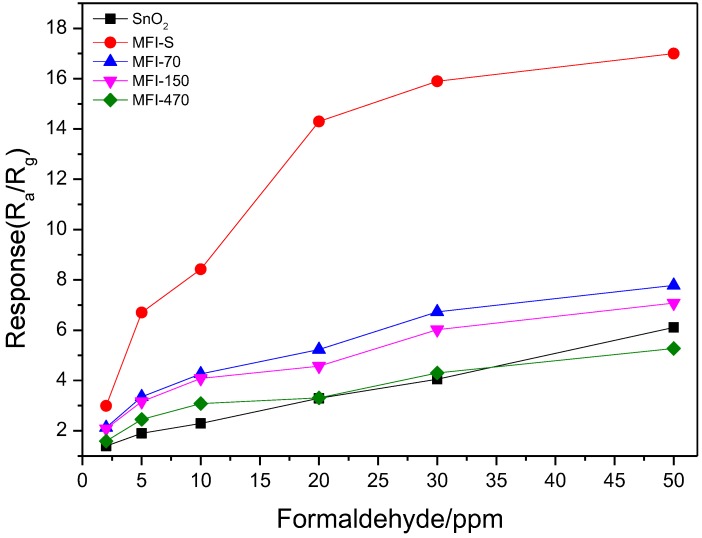
Sensors responses vs. concentration to formaldehyde.

Figure 7 illustrates the responses of SnO_2_ and ZSM-5 coated SnO_2_ gas sensors to formaldehyde in a concentration range of 2~50 ppm with a relative humidity 50% RH. We can see from Figure 7 that the response of MFI-S coated SnO_2_ sensors to formaldehyde is much higher than that of SnO_2_ in this concentration range and the response value reached 17 for 50 ppm formaldehyde. We can also find from the figure that the sensitivities (slope of the curves) for these sensors are different. The sensitivity in low concentration range is higher than that in high concentration range, especially for MFI-S coated SnO_2_ sensor. This is because the amount of gas molecules which adsorbed on sensitive materials increased with the rising of target gas concentration. The sensor’s response value increased rapidly, leading to a higher sensitivity. However, the adsorption saturates gradually with the increase of gas concentration, the response value increases slow subsequently, and the sensitivity become small. The gas saturation concentration is different for different sensing materials. The saturation concentration of the MFI-S coated SnO_2_ sensor is the highest in these sensors. With the increase of zeolitic SiO_2_/Al_2_O_3_ ratio, the response value and sensitivity of this type sensors decrease gradually.

**Figure 8:**
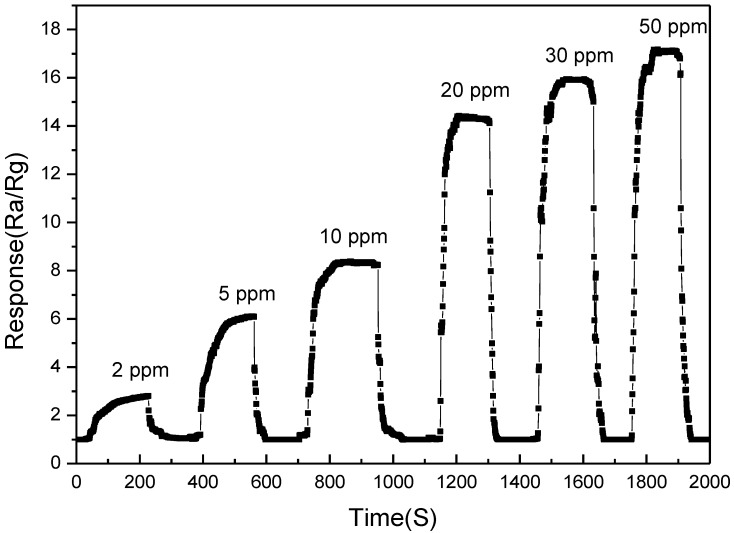
Transient response curves of the MFI-S coated SnO_2_ gas sensors to formaldehyde.

Figure 8 shows the transient response curves of the MFI-S coated SnO_2_ gas sensor to formaldehyde. The concentration of formaldehyde ranged from 2 ppm to 50 ppm, the operating temperature was 300 °C and the relative humidity was 50% RH.

**Figure 9:**
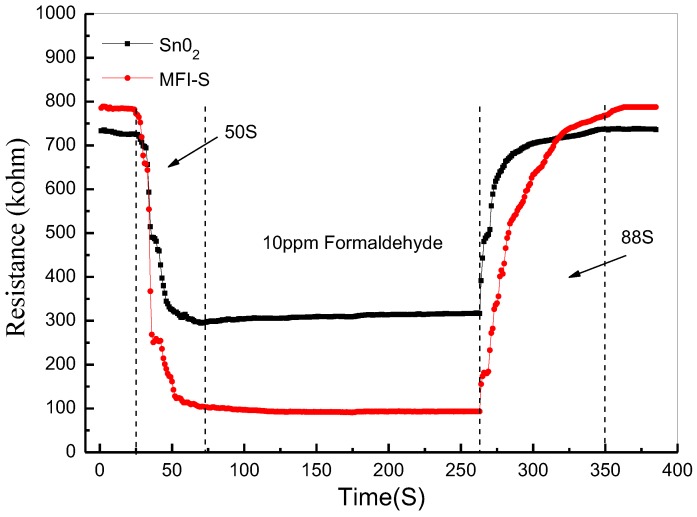
Transient response and recovery properties of SnO_2_ and MFI-S coated SnO_2_ sensors to 10 ppm formaldehyde.

Figure 9 gives the transient response and recovery properties of SnO_2_ and MFI-S coated SnO_2_ sensors to 10 ppm formaldehyde. The response and recovery times of MFI-S coated SnO_2_ sensor was a little longer than SnO_2_ sensor which were 50 s and 36 s, 88 s and 65 s, respectively. The response and recovery times were defined as the times reaching 90% of the final values. This may be due to the fact that the response value of the MFI-S coated SnO_2_ sensor is higher than the one of SnO_2_ sensor, resulting in longer response and recovery times. Meanwhile, the added zeolite layer delayed the arrival time of the target gas reach to the sensitive material (SnO_2_), and also affected the gas desorption time.

**Figure 10:**
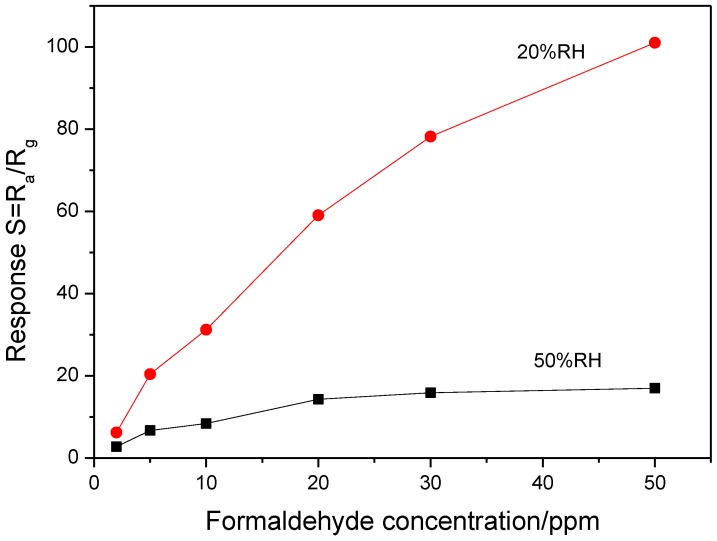
Responses of MFI-S coated sensor to 2–50 ppm formaldehyde in 20% RH and 50% RH.

Humidity is one of important influencing factors to properties of both zeolite and metal oxide. Figure 10 shows the response curves of MFI-S coated SnO_2_ sensor to different concentration formaldehyde in humidity of 20% RH and 50% RH, respectively. The response value of the MFI-S coated SnO_2_ sensors decreases with the increase of relative humidity, especially in high concentration range. This indicates that the additional water vapor seriously impacts the response value of the sensor to formaldehyde. The response values of the MFI-S coated SnO_2_ are 101 and 17 for 50 ppm formaldehyde in relative humidity of 20% RH and 50% RH, respectively.

We try to analyze this phenomenon as follows: The formaldehyde and acetone sensing processes can be described using the following reactions:

(1) $$\frac{1}{2}O_{2\left(g\right)}+e^{-}\to O_{\mathrm{ads}}^{-}$$

(2) $${\mathrm{HCHO}}_{\left(\mathrm{gas}\right)}+2O_{\mathrm{ads}}^{-}\to {\mathrm{CO}}_{2}+H_{2}O+2e^{-}$$

(3) $${\mathrm{CH}}_{3}{\mathrm{COCH}}_{3\left(\mathrm{gas}\right)}+8O_{\left(\mathrm{ads}\right)}^{-}\to 3{\mathrm{CO}}_{2}+3H_{2}O+8e^{-}$$

We can see from the reaction Equations (1)–(3) that electrons produce as the adsorbed oxygen ($O_{\mathrm{ads}}^{-})$ on the surface of the sensing materials reacts with formaldehyde and/or acetone vapor. However, the reaction can be driven back. With the increasing of humidity, more electrons take part in the reaction with water vapor [22,31], which will hinder the forward proceeding of the reaction, causing the increase of the sensor resistance (*R*_g_) and the decrease of the sensor response. On the other hand, humidity increases some water molecular adsorbed on the SnO_2_ surface and some of them adsorbed at a place in channel of zeolite which prevents the formaldehyde contact with SnO_2_ resulting a decreased response [36,37].

### 3.3. Gas Sensing Mechanism

The measurement results show that the response of MFI-S coated SnO_2_ sensors to formaldehyde increase, meanwhile, the response for acetone vapor decrease, indicating a good selectivity and sensitivity of the sensor to formaldehyde when the disturb gas was acetone. This effect is obvious when the coating materials ZSM-5 zeolite possess small particle size (MFI-S, 300 nm). The reason for an increased sensitivity of the sensor was analyzed as follows: The structure of MFI-S coated SnO_2_ is a layer of ZSM-5 zeolite filter layer covered outside of SnO_2_. When the sensors are placed in the formaldehyde atmosphere, some gas molecule can pass through the gaps of ZSM-5 layer and contact with SnO_2_, the others adsorption on internal acidic sites of zeolite. The gaps between crystalline also made gas easier pass through the ZSM-5 layer and contact with SnO_2_. The acidic sites of zeolites are the catalytic activity center. With the catalytic effect of the zeolite, the reducing gas formaldehyde is oxidized to generate water and other products, and some electrons are released [37]. The electrons are transported to the surface of SnO_2_, decreasing the resistance of the sensors. This result in an increased response value from 3.0 for SnO_2_ sensor to 11 for MFI-S coated SnO_2_ sensor. On the other hand, for large grain size ZSM-5 zeolite (~1 μm: MFI-70, MFI-150 and MFI-470), as the SiO_2_/Al_2_O_3_ ratio of the ZSM-5 increases, response value to formaldehyde decrease a little (4.4, 4.0 and 3.0 for MFI-70, MFI-150 and MFI-470). This may result from the number of acidic sites in ZSM-5 zeolites decreasing with the increase in SiO_2_/Al_2_O_3_ ratio, and the catalytic activity of the zeolite weakening [38]. At the same time, the crystalline size of ZSM-5 zeolites is a very important factor for increasing response value of the sensor to formaldehyde. The smaller the zeolite particle size is, the larger the specific surface area, and therefore the higher the catalytic activity [39].

It can be found that MFI-S (small grain size, low SiO_2_/Al_2_O_3_ ratio ZSM-5 zeolite) can effectively improve the response values of sensitive materials to formaldehyde by analyzing gas sensing properties of this type of sensor. Table 3 lists some similar structure of materials’ sensing properties to formaldehyde vapor. We find that response value of the MFI-S coated SnO_2_ sensor was higher than other sensors when comparing the sensing properties of the sensors in the list. The MFI-S could play an important role in this sensing process. Therefore, the MFI-S coated SnO_2_ sensor is promising to be used in the detection of low concentration of formaldehyde in the future, and this method (adding MFI-S layer) provides a possible new strategy to improve the response values of sensitive materials to formaldehyde gas detection.

The differences of the response values between the ZSM-5 coated SnO_2_ and SnO_2_ gas sensors to acetone were not obvious (Figure 6). The response values of the ZSM-5 coated SnO_2_ sensors to acetone appear different changes: compared with the SnO_2_ sensor, the response increases little for MFI-70, MFI-150 and MFI-470 coated SnO_2_ sensors, and decreases a small amount for MFI-S coated SnO_2_ sensors. Zeolite possesses both molecular sieve and catalytic properties. From the results of this experiment, catalytic property may play an important act at the large grain size zeolites (~1 μm: MFI-70, MFI-150 and MFI-470), resulting an increasing response; sieve property may play main role for small grain size zeolite (MFI-S, 300 nm), leading to a decreased response. The mechanism of zeolite in SnO_2_ sensing to VOC gases needs further investigation.

## 4. Conclusions

ZSM-5 zeolite with 300 nm grain size and ratio of SiO_2_/Al_2_O_3_ = 70 was prepared by using the template method. MFI-S and three other zeolites with grain size ~1 μm and ratio of SiO_2_/Al_2_O_3_ 70, 150 and 470 (MFI-70, MFI-150 and MFI-470) coated SnO_2_ gas sensors were fabricated, respectively. Their gas sensing properties were tested and compared with that of the SnO_2_ gas sensor. The results showed that the response value of MFI-S coated SnO_2_ gas sensors to formaldehyde vapor were obviously increased, and the response to acetone decreased compared with that of the pure SnO_2_ gas sensor, indicating an improved selectivity property. The other three ZSM-5 zeolites (MFI-70, MFI-150 and MFI-470, grain sizes all around 1 μm) coated with SnO_2_ sensors do not show much difference compared to the SnO_2_ sensor for the response properties to both formaldehyde and acetone. This indicates that the zeolite grain size in the ZSM-5 coated SnO_2_ sensors plays a crucial role. Humidity has a significant effect on the responses of the MFI-S zeolite coated SnO_2_ gas sensors. As humidity increases, the response of the sensor decreases obviously. The response and recovery times of the MFI-S coated SnO_2_ sensor was a little longer than the SnO_2_ sensor which was 50 s and 36 s, 88 s and 65 s, respectively. The grain sizes of ZSM-5 zeolites are a very important factor for increasing response value of the sensor to formaldehyde.

## Figures and Tables

**Table 1:** The SiO_2_/Al_2_O_3_ ratio and the grain size of four kinds of zeolites.

Zeolite	MFI-S	MFI-70	MFI-150	MFI-470
SiO_2_/Al_2_O_3_	70	70	150	470
Grain size	300 nm	~1 μm	~1 μm	~1 μm

**Table 2:** The response values of SnO_2_ gas sensor and ZSM-5 zeolites coated SnO_2_ sensors to gases with a relative humidity 45% RH.

Sensor	10 ppm Formaldehyde	10 ppm Acetone	10 ppm Benzene	10 ppm Toluene	10 ppm Ammonia
SnO_2_	3.5	5.0	1.4	1.6	1.4
MFI-S coated SnO_2_	11.0	3.5	1.4	1.9	1.4
MFI-70 coated SnO_2_	4.4	7.5	1.5	2.0	1.4
MFI-150 coated SnO_2_	4.0	6.8	1.4	1.7	1.4
MFI-470 coated SnO_2_	3.0	5.4	1.3	1.6	1.3

**Table 3:** The comparison on sensing properties of some similar structures sensors for formaldehyde gas detection.

Type	Structures of the Sensing Materials	Response Value (Concentration, Relative Humidity)	Operating Temperature (°C)	Response Time (s)	Recovery Time (s)
Obtained materials	MFI-S coated SnO_2_	11.0 (10 ppm, 45% RH)	300	50	88
Zeolite/metal oxide composite	ZnO and zeolitic imidazolate framework-8 core–shell heterostructures [16]	~13.0 (100 ppm,50% RH)	300	16	9
	Zeolitic imidazolate framework [40]	13.9 (100 ppm, <70% RH)	150	~100	~120
Hierarchical porous nanostructures	Hierarchical porous nanostructures of SnO_2_ [41]	~8.0 (10 ppm)	330	4.03	_
	Au@SnO_2_ core–shell structure [42]	2.9 (50 ppm, 50% RH)	room temperature	80	62
SnO_2_ and other oxides doped/heterostructures	NiO-doped SnO_2_ nanofiber [43]	6.3 (10 ppm)	200	50	80
	1D NiO-SnO_2_ nanofibers [44]	1.2 (20 ppm)	275	_	_
	SnO_2_/In_2_O_3_ hetero-nanofiber [9]	7.5 (10 ppm)	375	~50	~70
	MWCNTs-doped SnO_2_ [45]	~1.5 (10 ppm)	250	>100	>90
	Zn_2_SnO_4_/SnO_2_ cubes [46]	19.98 (20 ppm)	200	66	27
	SnO_2_/ZnO nanofibers [47]	~4.0 (50 ppm)	350	_	_
	3D center-hollow architecture and polyporous surface SnO_2_-ZnO composites [48]	~2 (10 ppm)	room temperature	_	_
	Cd-Doped TiO_2_-SnO_2_ [49]	32 (200 ppm)	320	25	17
	Antimony-Doped SnO_2_ nanoparticles [50]	~7.0 (10 ppm)	136	_	_

## References

[1] Kawamura K., Kerman K., Fujihara M., Nagatani N., Hashiba T., Tamiya E. (2005). Development of a novel hand-held formaldehyde gas sensor for the rapid detection of sick building syndrome. Sens. Actuators B Chem..

[2] Manoonkitiwongsa P.S., Schultz R.L. (2001). Proper nomenclature of formaldehyde and paraformaldehyde fixatives for histochemistry. Histochem. J..

[3] Koziel J.A., Noah J., Pawliszyn J. (2001). Field sampling and determination of formaldehyde in indoor air with solid-phase microextraction and on-fiber derivatization. Environ. Sci. Technol..

[4] Suzuki Y., Nakano N., Suzuki K. (2003). Portable sick house syndrome gas monitoring system based on novel colorimetric reagents for the highly selective and sensitive detection of formaldehyde. Environ. Sci. Technol..

[5] Lee J.-H. (2009). Gas sensors using hierarchical and hollow oxide nanostructures: Overview. Sens. Actuators B Chem..

[6] DU H.-Y., Wang J., WU N. (2009). Improvement of gas sensing-characteristics of formaldehyde gas sensor by doping. Instrum. Tech. Sens..

[7] Zheng W., Lu X., Wang W., Dong B., Zhang H., Wang Z., Xu X., Wang C. (2010). A rapidly responding sensor for methanol based on electrospun In_2_O_3_-SnO_2_ nanofibers. J. Am. Ceram. Soc..

[8] Chiu H.-C., Yeh C.-S. (2007). Hydrothermal synthesis of SnO_2_ nanoparticles and their gas-sensing of alcohol. J. Phys. Chem. C.

[9] Du H.-Y., Wang J., Su M., Yao P., Zheng Y., Yu N. (2012). Formaldehyde gas sensor based on SnO_2_/In_2_O_3_ hetero-nanofibers by a modified double jets electrospinning process. Sens. Actuators B Chem..

[10] Zhang W., Tian J., Wang Y.A., Fang X., Huang Y., Chen W., Liu Q., Zhang D. (2014). Single porous SnO_2_microtubes templated from papilio maacki bristles: New structure towards superior gas sensing. J. Mater. Chem. A.

[11] Huang J., Li G., Huang Z., Huang X., Liu J. (2006). Temperature modulation and artificial neural network evaluation for improving the CO selectivity of SnO_2_ gas sensor. Sens. Actuators B Chem..

[12] Liewhiran C., Tamaekong N., Wisitsoraat A., Tuantranont A., Phanichphant S. (2013). Ultra-sensitive H_2_ sensors based on flame-spray-made Pd-loaded SnO_2_ sensing films. Sens. Actuators B Chem..

[13] Jang B.-H., Landau O., Choi S.-J., Shin J., Rothschild A., Kim I.-D. (2013). Selectivity enhancement of SnO_2_ nanofiber gas sensors by functionalization with Pt nanocatalysts and manipulation of the operation temperature. Sens. Actuators B Chem..

[14] Yimlamai I., Niamlang S., Chanthaanont P., Kunanuraksapong R., Changkhamchom S., Sirivat A. (2011). Electrical conductivity response and sensitivity of ZSM-5, Y, and mordenite zeolites towards ethanol vapor. Ionics.

[15] Hernández P.T., Hailes S., Parkin I. (2017). Hydrocarbon detection with metal oxide semiconducting gas sensors modified by overlayer or admixture of zeolites Na-A, H-Y and H-ZSM-5. Sens. Actuators B Chem..

[16] Tian H., Fan H., Li M., Ma L. (2015). Zeolitic imidazolate framework coated ZnO nanorods as molecular sieving to improve selectivity of formaldehyde gas sensor. ACS Sens..

[17] Zheng Y., Li X., Dutta P.K. (2012). Exploitation of unique properties of zeolites in the development of gas sensors. Sensors.

[18] Sahner K., Hagen G., Schönauer D., Reiß S., Moos R. (2008). Zeolites—Versatile materials for gas sensors. Solid State Ion..

[19] Xu X., Wang J., Long Y. (2006). Zeolite-based materials for gas sensors. Sensors.

[20] Binions R., Afonja A., Dungey S., Lewis D.W., Parkin I.P., Williams D.E. (2011). Discrimination effects in zeolite modified metal oxide semiconductor gas sensors. IEEE Sens. J..

[21] Varsani P., Afonja A., Williams D.E., Parkin I.P., Binions R. (2011). Zeolite-modified WO_3_ gas sensors—Enhanced detection of NO_2_. Sens. Actuators B Chem..

[22] Vilaseca M., Coronas J., Cirera A., Cornet A., Morante J., Santamaria J. (2008). Development and application of micromachined Pd/ SnO_2_ gas sensors with zeolite coatings. Sens. Actuators B Chem..

[23] Sahner K., Schönauer D., Moos R., Matam M., Post M.L. (2006). Effect of electrodes and zeolite cover layer on hydrocarbon sensing with p-type perovskite SrTi_0.8_Fe_0.2_O_3−δ_ thick and thin films. J. Mater. Sci..

[24] Fukui K., Nishida S. (1997). CO gas sensor based on Au–La_2_O_3_ added SnO_2_ ceramics with siliceous zeolite coat. Sens. Actuators B Chem..

[25] Sahner K., Schönauer D., Kuchinke P., Moos R. (2008). Zeolite cover layer for selectivity enhancement of p-type semiconducting hydrocarbon sensors. Sens. Actuators B Chem..

[26] Jadsadapattarakul D., Thanachayanont C., Nukeaw J., Sooknoi T. (2010). Improved selectivity, response time and recovery time by [010] highly preferred-orientation silicalite-1 layer coated on SnO_2_ thin film sensor for selective ethylene gas detection. Sens. Actuators B Chem..

[27] Yang P., Lau C., Liang J.Y., Lu J.Z., Liu X. (2007). Zeolite-based cataluminescence sensor for the selective detection of acetaldehyde. Luminescence.

[28] Haw J.F. (2002). Zeolite acid strength and reaction mechanisms in catalysis. Phys. Chem. Chem. Phys..

[29] Konno H., Tago T., Nakasaka Y., Watanabe G., Masuda T. (2014). Characterization and catalytic performance of modified nano-scale ZSM-5 for the acetone-to-olefins reaction. Appl. Catal. A Gen..

[30] Viswanadham N., Kamble R., Singh M., Kumar M., Dhar G.M. (2009). Catalytic properties of nano-sized ZSM-5 aggregates. Catal. Today.

[31] Vilaseca M., Coronas J., Cirera A., Cornet A., Morante J., Santamarıa J. (2003). Use of zeolite films to improve the selectivity of reactive gas sensors. Catal. Today.

[32] Xu X., Wang J., Long Y. (2005). Nano-tin dioxide/NaY zeolite composite material: Preparation, morphology, adsorption and hydrogen sensitivity. Microporous Mesoporous Mater..

[33] Xu D. (2011). Preparation and Investigation on Properties of Hierarchical Porous Nano-Molecular Sieves. Ph.D. Thesis.

[34] Du H.-Y., Wang J., Yu P., Yu N.-S., Sun Y.-H., Tian J.-L. (2014). Investigation of gas sensing materials tin oxide nanofibers treated by oxygen plasma. J. Nanopart. Res..

[35] Yang H. (2007). The Effect of Crystal Size on Selective Catalytic Reduction of NOx by Acetylene over Zeolites. Master’s Thesis.

[36] Vilaseca M., Coronas J., Cirera A., Cornet A., Morante J.R., Santamaria J. (2007). Gas detection with SnO_2_ sensors modified by zeolite films. Sens. Actuators B Chem..

[37] Trimboli J., Dutta P.K. (2004). Oxidation chemistry and electrical activity of Pt on titania: Development of a novel zeolite-filter hydrocarbon sensor. Sens. Actuators B Chem..

[38] Xiao L. (2014). Preparation and Catalytic Properties of SnO2 with Different Morphologies. Master’s Thesis.

[39] Shetti V.N., Kim J., Srivastava R., Choi M., Ryoo R. (2008). Assessment of the mesopore wall catalytic activities of MFI zeolite with mesoporous/microporous hierarchical structures. J. Catal..

[40] Chen E.X., Yang H., Zhang J. (2014). Zeolitic imidazolate framework as formaldehyde gas sensor. Inorg. Chem..

[41] Xu K., Zeng D., Tian S., Zhang S., Xie C. (2014). Hierarchical porous SnO_2_ micro-rods topologically transferred from tin oxalate for fast response sensors to trace formaldehyde. Sens. Actuators B Chem..

[42] Chung F.-C., Wu R.-J., Cheng F.-C. (2014). Fabrication of a Au@SnO_2_ core–shell structure for gaseous formaldehyde sensing at room temperature. Sens. Actuators B Chem..

[43] Zheng Y., Wang J., Yao P. (2011). Formaldehyde sensing properties of electrospun NiO-doped SnO_2_ nanofibers. Sens. Actuators B Chem..

[44] Tang W., Wang J. (2016). 1D NiO-SnO_2_ heterojunction nanofibers as acetone sensor. KnE Mater. Sci..

[45] Wang J., Liu L., Cong S.-Y., Qi J.-Q., Xu B.-K. (2008). An enrichment method to detect low concentration formaldehyde. Sens. Actuators B Chem..

[46] Tang W., Wang J., Yao P., Li X. (2013). A microscale formaldehyde gas sensor based on Zn_2_Sno_4_/Sno_2_ and produced by combining hydrothermal synthesis with post-synthetic heat treatment. J. Mater. Sci..

[47] Tang W. (2017). Sensing mechanism of SnO_2_/ZnO nanofibers for CH_3_OH sensors: Heterojunction effects. J. Phys. D Appl. Phys..

[48] Jiang J., Shi L., Xie T., Wang D., Lin Y. (2018). Study on the gas-sensitive properties for formaldehyde based on SnO_2_-ZnO heterostructure in uv excitation. Sens. Actuators B Chem..

[49] Zeng W., Liu T., Wang Z., Tsukimoto S., Saito M., Ikuhara Y. (2009). Selective detection of formaldehyde gas using a cd-doped TiO_2_-SnO_2_ sensor. Sensors.

[50] Wang Y.D., Djerdj I., Antonietti M., Smarsly B. (2008). Polymer-assisted generation of antimony-doped SnO_2_ nanoparticles with high crystallinity for application in gas sensors. Small.

